# Thoracoscopic resection of a vagal schwannoma in the superior mediastinum: A case report

**DOI:** 10.3892/ol.2014.2116

**Published:** 2014-05-07

**Authors:** ZHIQIANG WU, MIN SHI, HONGLI WAN, WEI GAO, HUIPING LIU, ZHANPENG WANG, QINGXIN LI

**Affiliations:** 1 Department of Thoracic Surgery, Lanzhou General Hospital, Lanzhou Command, Lanzhou, Gansu 730050, P.R. China; 2 Department of Pathology, Lanzhou General Hospital, Lanzhou Command, Lanzhou, Gansu 730050, P.R. China; 3 Department of Gynecology and Obstetrics, Lanzhou General Hospital, Lanzhou Command, Lanzhou, Gansu 730050, P.R. China

**Keywords:** thoracoscopic surgery, schwannoma, neurilemomas, vagus nerve, mediastinum

## Abstract

Neurogenic tumors are the most common type of mediastinal tumor and constitute the majority of neoplasms of the posterior mediastinum. Schwannomas originating from the intrathoracic vagus nerve are extremely rare. The present study describes the case of a 58-year-old man with a large vagal schwannoma in the left superior mediastinum. A large tumor with a round shape was identified in the left superior mediastinum. The tumor originated from and encased the vagus nerve. Using video-assisted thoracoscopic surgery, the tumor was completely excised with amputation of the vagus nerve encased within in the tumor. One year post-surgery, the patient was free of recurrence with no symptoms other than hoarseness.

## Introduction

Neurogenic tumors are the most common type of mediastinal tumor and constitute the majority of neoplasms of the posterior mediastinum (1). Neurogenic tumors derive from the cells of the nerve sheath or from the ganglionic cells of the spinal ganglia and of the autonomic, paraganglionic and parasympathetic systems (2). Schwannoma is a type of benign nerve sheath tumour which is ofen located in the head, neck and extremities, and mostly asymptomatic. However, occasionally schwannoma may present with symptoms of compression of the neighboring structures. The treatment of choice is surgical excision, with good prognosis. However, schwannoma, which arises from the intrathoracic vagus nerve, is rare. The present study describes a rare case of a vagal schwannoma in the left superior mediastinum, which was resected en bloc using video-assisted thoracoscopic surgery (VATS). The patient provided written informed consent.

## Case report

### Case presentation

**Figure 1:**
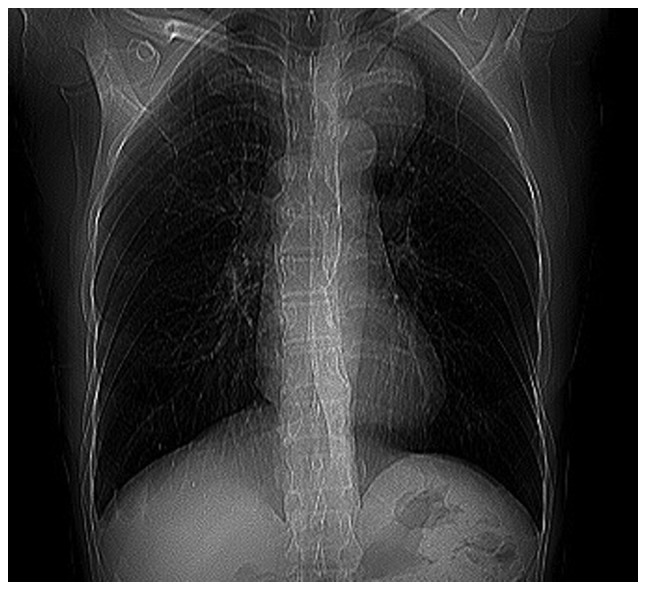
Posteroanterior chest roentgenogram demonstrating a left superior lung field mass protruding from the mediastinum.

**Figure 2:**
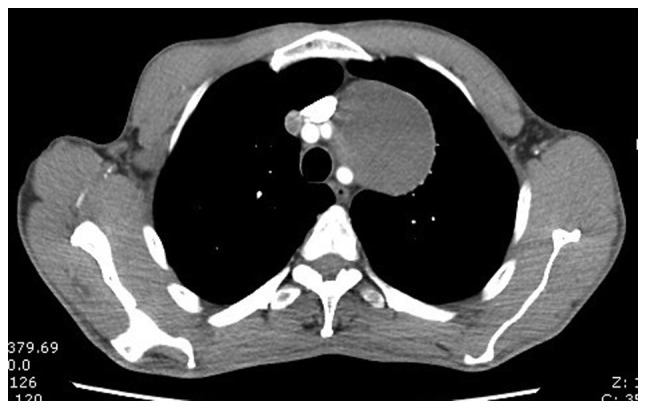
Contrast-enhanced computed tomography of the chest showing the circumscribed mass identifiable in the left superior mediastinum with a smooth and clear margin, and measuring 78×66×59 mm.

A 58-year old male presented with chest pain and hoarseness for two months. The patient denied symptoms, including fever, dyspnea, palpitation, hemoptysis, dysphagia and muscle weakness. The patient’s past medical history was not significant. Physical examination and laboratory test results showed no significant abnormalities, including those in carcinoembryonic antigen, α-fetoprotein and prostate specific antigen levels. A chest roentgenogram revealed a well-defined mass located at the left superior lung field, protruding from the mediastinum (Fig. 1). Contrast-enhanced computed tomography of the chest showed a sharply demarcated, circumscribed mass, ~78×66×59 mm in size, in the left superior mediastinum (Fig. 2).

### Surgery and histological analysis

**Figure 3:**
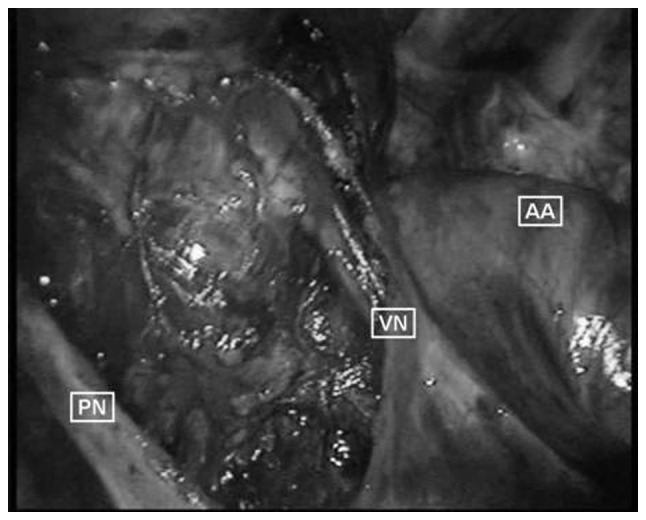
Intraoperative view of the schwannoma showing the tumor originating from, and encasing the vagus nerve. VN, vagus nerve; PN, phrenic nerve; AA, aortic arch.

**Figure 4:**
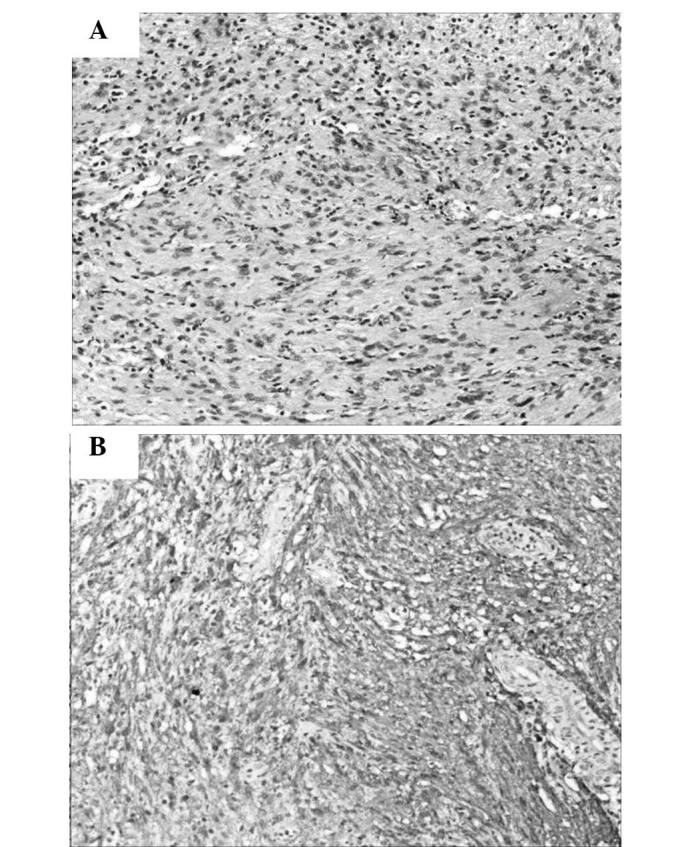
(A) Histopathology of the mediastinal vagus nerve schwannoma (stain, hematoxylin and eosin; magnification, ×200). (B) Strong positivity for S-100 protein in the schwannoma cells (magnification, ×200).

The patient underwent left-sided VATS. Intraoperatively, a large tumor with a round shape was identified in the left superior mediastinum. The left phregnic nerve crossed the surface of the mass and the tumor was originating from, and encasing, the vagus nerve (Fig. 3). The mass was located at the anterior and superior to the aortic arch, and was attached to the left subclavian artery, left common carotid artery, left innominate vein and superior vena cava. The tumor was completely excised through amputation of the vagus nerve encased in the mass. Grossly, the mass had a complete envelop and contained dark-colored hydatid fluid. Histologically, the tumor contained spindle cells with strong positivity for S-100 protein and was diagnosed as schwannoma of the vagus nerve (Fig. 4).

### Follow-up

The patient’s postoperative recovery was uneventful and the patient was discharged on the seventh postoperative day. The patient was followed up at six month intervals for 18 months. At the one-year follow-up, the patient was tumor- and symptom-free, but presented with hoarseness.

## Discussion

Schwannoma, also termed neurilemmoma, is a type of benign nerve sheath tumor arising from Schwann cells. It is the most common neurogenic tumor of the chest and approximately 10% of schwannomas originate from the vagus nerve (3–5). In 1935, Stout (6) first designated vagal tumors of nerve sheath origin as ‘neurilemmomas’. The tumor may occur at all ages and does not show a gender preference. Schwannoma is asymptomatic in the majority of cases; however, a number of symptoms, including chest pain, dysphagia, coughing and hoarseness, to varying degrees, may occur due to compression of the neighboring organs (7,8). Hoarseness may occur when the tumor is influenced by the recurrent laryngeal nerve, as was shown in the present case. Schwannomas of the vagus nerve are almost twice as likely to be located on the left than on the right, as the recurrent laryngeal nerve arises lower in the thoracic cavity on the left side and the left nerve trunk is thicker (1,8–11).

Surgical resection is recommended for mediastinal neurogenic tumors and thoracoscopic surgery is preferred due to its less invasive nature, which is beneficial when resecting sharply marginated masses, as in the present case. Although certain studies have proposed that VATS was contraindicated in tumors larger than 6 cm (9,10), Yamaguchi *et al* (12) reported that VATS was capable of excising a neurogenic tumor of the thorax as large as 7 cm in diameter, with no complications. In the present case, the tumor was large and attached to the great vessels; however, it was partly cystic and the tumor was resected using VATS with tumor incision and hydatid fluid outflow. When the tumor encased the vagal nerve, enucleation of the schwannoma from the vagal nerve is difficult and amputation of the nerve is unavoidable with sacrifice of the recurrent laryngeal nerve branch, as was shown in the present case. The patient should be closely observed for cardiac rhythm abnormalities, as severe bradycardia or asystole may develop during removal of the tumor (13,14).

The specific diagnosis of schwannoma requires pathological examination. In the present case, microscopic examination revealed spindle cells in fascicles in a loose stroma. If atypia, mitoses, pleomorphism and necrosis are identified, malignant schwannoma should be considered in the diagnosis, although they are extremely rare (4).

The prognosis of schwannoma of the vagus nerve following complete resection of the tumor appears to be satisfactory. The patient described in the present case was free of recurrence with no symptoms at the one-year follow-up; however, long-term survival should be assessed.
